# A miniPCR-Duplex Lateral Flow Dipstick Platform for Rapid and Visual Diagnosis of Lymphatic Filariae Infection

**DOI:** 10.3390/diagnostics11101855

**Published:** 2021-10-05

**Authors:** Achinya Phuakrod, Witsaroot Sripumkhai, Wutthinan Jeamsaksiri, Pattaraluck Pattamang, Sumat Loymek, Paul J. Brindley, Patsharaporn T. Sarasombath, Sirichit Wongkamchai

**Affiliations:** 1 Department of Microbiology, Faculty of Medicine Siriraj Hospital, Mahidol University, Bangkok 10700, Thailand; achinya.phu@mahidol.ac.th; 2 Thai Microelectronic Center, National Electronics and Computer Technology Center, Thailand Science Park, Pathum Thani 12110, Thailand; Witsaroot.Sripumkhai@nectec.or.th (W.S.); Wutthinan.Jeamsaksiri@nectec.or.th (W.J.); Pattaraluck.Pattamang@nectec.or.th (P.P.); 3 Office of Disease Prevention and Control, Region 12, Department of Disease Control, The Ministry of Public Health, Songkhla 9000, Thailand; sumatloimej@gmail.com; 4 Immunology & Tropical Medicine & Research Center for Neglected Diseases of Poverty, Department of Microbiology, School of Medicine & Health Sciences, George Washington University, Washington, DC 20037, USA; pbrindley@email.gwu.edu; 5 Department of Parasitology, Faculty of Medicine Siriraj Hospital, Mahidol University, Bangkok 10700, Thailand

**Keywords:** *Brugia malayi*, *Wuchereria bancrofti* DLFD, duplex lateral flow dipstick, lymphatic filariasis, miniPCR

## Abstract

Lymphatic filariasis (LF) is a neglected major tropical disease that is a leading cause of permanent and long-term disability worldwide. Significant progress made by the Global Programme to Eliminate Lymphatic Filariasis (GPELF) has led to a substantial decrease in the levels of infection. In this limitation, DNA detection of lymphatic filariae could be useful due to it capable of detecting low level of the parasites. In the present study, we developed a diagnostic assay that combines a miniPCR with a duplex lateral flow dipstick (DLFD). The PCR primers were designed based on the *Hha*I and *Ssp*I repetitive noncoding DNA sequences of *Brugia malayi* and *Wuchereria bancrofti*, respectively. The limits of detection and crossreactivity of the assay were evaluated. In addition, blood samples were provided by Thais living in a brugian filariasis endemic area. The miniPCR-DLFD assay exhibited a detection limit of 2 and 4 mf per milliliter (mL) of blood for *B. malayi* as well as *W. bancrofti*, respectively, and crossamplification was not observed with 11 other parasites. The result obtained from the present study was in accordance with the thick blood smear staining for the known cases. Thus, a miniPCR-DLFD is an alternative tool for the diagnosis of LF in point-of-collection settings with a modest cost (~USD 5) per sample.

## 1. Introduction

Lymphatic filariasis (LF) caused by *W. bancrofti*, *B. malayi* and *B. timori*, is one of the most neglected tropical diseases. LF is a mosquito-borne disease and remains a major public health concern [1,2]. The parasites can cause clinical complications of lymphedema, hydrocele, and lead to elephantiasis, making it the second leading disabling disease in the world [3]. According to the GPELF established by the World Health Organization (WHO), a mass drug administration (MDA) was given to residents of LF endemic areas [4]. Diagnostic tests play important roles to ensure that an MDA program is on track to achieve its goal and determine when that goal is achieved and whether the program can be stopped [5].

The classical diagnosis of lymphatic filariasis is based on the microscopic examination of microfilariae using thick blood smear staining. In addition, a variety of methods such as antigen detection, antibody detection, and molecular diagnosis have been developed to improve diagnostic performance and utility [6,7,8]. An immunochromatographic test (ICT) for the detection of *W. bancrofti*-circulating antigen is available commercially [8]. ICTs are easy to perform, and their results are easily interpreted. Nevertheless, filarial antigenemia that persists for years, including after treatment, limits the value of *W. bancrofti* antigen detection, as evidenced by follow-up studies posttreatment [9,10,11]. Several articles have reported tests that can detect circulating antigens from *B. malayi* in human blood. However, those tests have not yet been independently verified, and none are commercially available [3,12,13,14,15,16]. Antifilarial IgG4 antibody detection has also been developed [6,7,17], and it is a useful addition to the limited array of brugian filariasis diagnostic tools available. Measuring antibodies returns a cumulative/longitudinal history of the infection. However, antibody tests cannot distinguish between bancroftian and brugian filariasis, limiting their usefulness in areas where these infections are coendemic. In several countries that have completed multiple rounds of mass drug administration, dramatic reductions in both microfilaremia and antigenemia levels have been observed [18].

Regarding the molecular diagnosis for LF, PCR assays have the advantages of active infection detection and causative species identification with reliable results [19,20]. The disadvantages of the standard PCR assays include that they are time-consuming, require dedicated laboratory instruments and reagents, and are available at only certain facilities. Several compact PCR-based methods and devices have been validated for use for point-of-collection detections [21,22]. The miniPCR thermocycler represents an attractive and affordable device with the potential for use at collection sites in endemic-country settings. This user-friendly and portable instrument is commercially available at a modest price [21,22]. However, the miniPCR still requires gel-analysis steps involving gel electrophoresis and imaging. To obviate these latter steps, several studies have proposed the use of a PCR-nucleic acid lateral flow immunochromatographic assay (PCR-NALFIA) using a lateral flow dipstick (LFD) [22,23]. LFD-based assays can detect specific DNA products in as little as 10 min [22,23].

The present study proposed for the first time to develop a miniPCR assay coupled with a duplex lateral flow dipstick (DLFD) for rapid and visual detection of both *B. malayi* and *W. bancrofti* DNA in human blood samples.

## 2. Materials and Methods

### 2.1. Ethics Approval

Before commencement of the study, ethics approval was obtained from the Ethics Committee of the Faculty of Medicine Siriraj Hospital, Mahidol University (approval number, Si129/2016). The research also fully complied with the ethical principles and guidelines for human experimentation issued by the National Research Council of Thailand. The formal consent of the participants was obtained verbally.

### 2.2. Microscopic Detection of Microfilariae in Blood Samples Using Giemsa Staining

Giemsa staining was performed according to the standard WHO procedure [24]. Briefly, thick blood smear slides were prepared from 50 µL of ethylenediaminetetraacetic acid (EDTA) blood samples. After drying overnight, the slides were immersed in freshly prepared working Giemsa stain for 45–60 min. Then, it was removed and rinsed by dipping 3–4 times in the Giemsa buffer. After air-drying, the slides were examined under a microscope (40×) for the detection of mf.

### 2.3. Development of a miniPCR Assay

#### 2.3.1. Extraction of DNA from Blood Samples

DNA was extracted from 50 µL of EDTA blood samples using the High Pure PCR Template Preparation Kit (Roche Diagnostics GmbH, Penzberg, Germany) according to the manufacturer’s protocol. Following extraction, the DNA was eluted in 100 µL of elution buffer and stored at −20 °C until use. The DNA concentration was determined using a Nanodrop 1000 spectrophotometer (Thermo Fisher Scientific Inc, Waltham, MA, USA).

#### 2.3.2. Primer Design and Optimization of Standard PCR Condition

A pair of primers for *B. malayi* was designed based on the alignment of different regions of the *Hha*I repetitive noncoding DNA sequences of *B. malayi*. The sequences of the forward and reverse primers are forward 5′ CTTCATTAGACAAGGATATTGGTTC and reverse 5′ GACAACACAATACACGACCAG. The sequences were obtained from the National Center for Biotechnology Information database (www.ncbi.nlm.nih.gov; accession number M12691.1; position 132–277). The sequences of forward and reverse primers recognized a 145 bp region of the *HhaI* repetitive noncoding DNA sequences of *B. malayi.* A BLAST search (https://www.ncbi.nlm.nih.gov/BLAST/, accessed on 15 March 2020)) was performed to check the specificity of the primer to the target DNA.

A pair of primers was designed based on the alignment of different regions of the *SspI* repetitive noncoding DNA sequences of *W. bancrofti*. The sequences of the forward and reverse primers are forward 5′ CAAAGTAGCGTAAGGGAATTG and reverse 5′ CCCTCACTTACCATAAGACAAC. The Sequences were obtained from the National Center for Biotechnology Information database (www.ncbi.nlm.nih.gov; accession number L20344.1; position 13-195. The sequences of forward and reverse primers recognized a 182 bp region of the *Ssp*I repetitive noncoding DNA sequences of *W. bancrofti.* A BLAST search (https://www.ncbi.nlm.nih.gov/BLAST/, accessed on 15 March 2020) was performed to check the specificity of the primer to the target DNA.

#### 2.3.3. Optimization of Standard PCR Assay

To obtain the optimal PCR condition, a gradient PCR was performed using the Veriti 96-Well Thermal Cycler (Applied Biosystems, Thermo Fisher Scientific Inc, Waltham, MA, USA) with the annealing temperature ranging from 55 to 62 °C for both sets of primers (*Hha*I and *Ssp*I). The PCR condition of *Hha*I primer set for *B. malayi* as well as that of *Ssp*I primer set for *W. bancrofti*, were conducted in a volume of 20 µL consisting of 10 µL of PCR master mix, 0.2 µM of each forward and reverse primer, 7.2 µL of dH_2_O, and 2 µL of DNA template.

The PCR assay for *B. malayi* and *W. bancrofti* was performed in a separate tube for each primer sets. The optimized amplification conditions included an activation step at 95 °C for 5 min, followed by a 30-step amplification of 30 s at 95 °C, 30 s at 56 °C, and 30 s at 70 °C, with the last step at 70 °C for 5 min with the PCR amplifications annealing temperature at 56°C. A volume of 20 µL was created, consisting of 10 µL of PCR master mix (Quantabio; Qiagen Beverly, MA, USA), 0.2 µM of each forward and reverse primer, 7.2 µL of dH_2_O, and 2 µL of DNA template. Nuclease-free water was used as a negative control. DNA of *B. malayi* and *W. bancrofti* were used as positive controls.

#### 2.3.4. The Specificity of the Primer

The specificity of each primer set was evaluated. The primers of *B. malayi* were performed using both annealing temperatures of 56 °C and 57 °C with *W. bancrofti* DNA as a template, whereas the primers of *W. bancrofti* were performed using *B. malayi* DNA as a template. There was no amplification of nonspecific bands at both annealing temperatures for both primer sets.

#### 2.3.5. The miniPCR Assay for Amplification of *W. bancrofti* and *B. malayi* DNA

**Figure 1:**
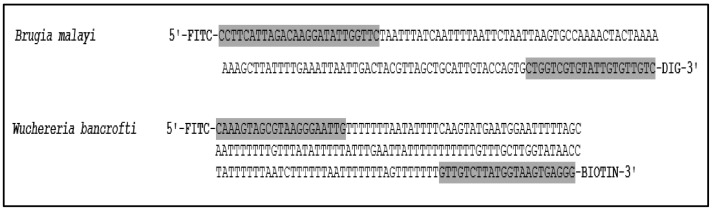
Alignment of *Hha*I repetitive noncoding DNA sequence of *Brugia malayi* (GenBank accession number M12691.1; position 132-277) and *Ssp*I repetitive noncoding DNA sequence of *Wuchereria bancrofti* (GenBank accession number L20344.1, positions 13-195). Gray areas indicate primers. For the DLFD, the *Hha*I and *Ssp*I primers were labeled with fluorescein isothiocyanate (FITC) and digoxin (DIG), respectively.

The PCR assays for *B. malayi* and *W. bancrofti* were performed using a miniPCR instrument (DBA miniPCR bio; Amplyus LLC., Cambridge, MA, USA), employing the same reagents and conditions used for the standard PCR, as noted above, except for the primer sets. The 5′ ends of the designed forward primers of *Hha*I and *Ssp*I were labeled with fluorescein isothiocyanate (FITC). The 5′ ends of reverse primers of *Hha*I and *Ssp*I were labeled with digoxin (DIG) and biotin, respectively. The sequences of the primers and their corresponding amplicons are presented in Figure 1. Each set of primer (*Hha*I and *Ssp*I) was performed in a separate tube.

### 2.4. Construction of Duplex Lateral Flow Dipstick (DLFD) for Detection of W. Bancrofti and B. Malayi DNA

**Figure 2:**
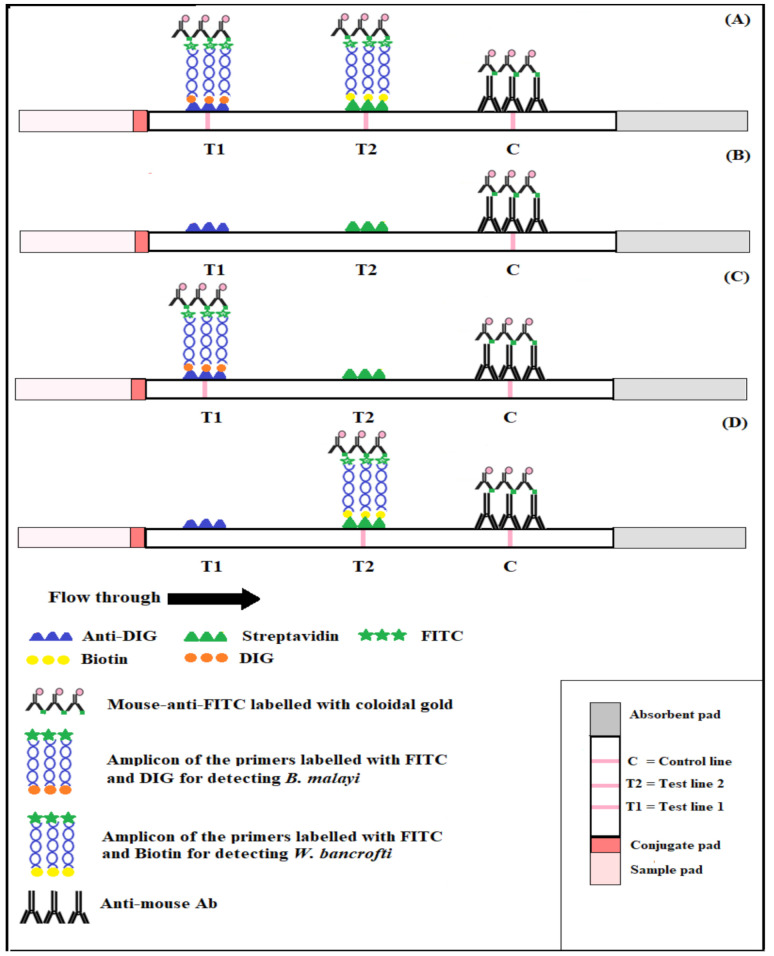
Schematic illustration of the duplex lateral flow dipstick (DLFD) for two target detections. T1 = test line 1 for detecting *Brugia malayi*; T2 = test line 2 for detecting *Wuchereria bancrofti*; C = control line. (**A**) = positive control; (**B**) = negative control; (**C**) = positive for *W. bancrofti*; (**D**) = positive for *B. malayi*.

A DLFD is composed of four parts: the sample pad, conjugate pad, nitrocellulose membrane, and absorbent pad. The sample pad is pretreated with buffer, and it can offer suitable pH and ion strength for detection. The conjugate pad is used for the storage of reporter molecules (colloidal gold conjugated mouse anti-FITC). For the development of our DLFD, anti-digoxin (anti-DIG; Test line 1) and streptavidin (Test line 2) were sprayed on the nitrocellulose membrane to create test zones using the IsoFlow Reagent Dispenser (Imagene Technology, Inc., Hanover, NH, USA), whereas anti-mouse antibody was sprayed on the nitrocellulose membrane to form a control zone (control line) by the AirJet Quanti 3000 Nanoliter aerosol dispenser (BioDot, Inc., Irvine, CA, USA). The membrane was then dried at 37 °C for 12 h. The nitrocellulose membrane was attached to the central part of an adhesive plate. The lateral flow dipstick was then assembled. Figure 2 shows a schematic illustration of the DLFD.

#### 2.4.1. Optimization of Anti-DIG and Streptavidin Concentration

Various concentrations of anti-DIG (0.5 µg/strip, 0.75 µg/strip, or 1 µg/strip; Test line (1) were sprayed on the nitrocellulose membrane to optimize the concentration of anti-DIG, using the IsoFlow Reagent Dispenser (Imagene Technology, Inc., Hanover, NH, USA). The concentrations were at 1 µL per mm. The lateral flow dipstick was then assembled. Various concentrations of streptavidin (i.e., 0.5 µg/strip, 0.75 µg/strip, or 1 µg/strip; Test line (2) were sprayed on the nitrocellulose membrane to optimize the concentration of streptavidin using the AirJet Nanoliter aerosol dispenser (BioDot, Inc., Irvine, CA, USA). The assay was performed using positive (DNA of *B. malayi*) and negative (dH_2_O) controls.

#### 2.4.2. DLFD for Detection of Amplification Products

To detect the amplification products of each sample, 1 µL of amplification product from each set of primers (specific to *Hha*I and *Ssp*I) was added into a well of the 96-well plate containing 100 µL of sample buffer. The dipstick was placed into the well vertically, and the reaction was read within 5–10 min. The appearance of positive pink-colored lines was observed using the naked eye on both the test and control lines. For negative results, the pink-colored line was apparent solely on the control line.

### 2.5. The miniPCR-DLFD Specificity

To verify the specificity of the miniPCR-DLFD-based detection platform, genomic DNA was isolated from 11 other parasites—*Trichinella spiralis*, *Angiostrongylus cantonensis*, *Gnathostoma spinigerum*, *Enterobius vermicularis*, *Necator americanus*, *Taenia solium*, *Plasmodium falciparum*, *Litomosoides sigmodontis*, *Brugia pahangi*, *Dirofilaria immitis* and *D. repens*—and used to measure off-target PCR amplification by the miniPCR-DLFD. The amount of DNA used for the miniPCR of each parasite was 20 ng/reaction. For other filariae i.e., *B. timori*, *Mansonella* spp., *Loa loa,* and *Onchocerca volvulus*, unfortunately, we were unable to obtain the parasite DNAs. The sequence similarity of *B. malayi* and *W. bancrofti* primers were compared with sequences of the closely related filaria species i.e., *B. timori, Masonella spp*., *Loa,* and *Onchocerca spp*. (*O. volvulus*) using the nucleotide BLAST program (National Center for Biotechnology Information, NCBI, Bethesda, MD, USA; Available online: www.ncbi.nlm.nih.gov: accessed on 15 September 2021).

### 2.6. Detection Limit of miniPCR-DLFD

To study the detection limit of the miniPCR-DLFD, mf of *B. malayi* as well as mf of *W. bancrofti* was spiked into EDTA blood samples obtained from healthy subjects, as listed in Table 1. For each species, 4 sets of the samples were prepared (sample IDs 1–24 for *B. malayi* and sample IDs 25–48 for *W. bancrofti*). The DNA of *B. malayi*, *W. bancrofti*, as well as negative blood samples, were used as positive and negative controls. DNA that had been extracted from the blood samples underwent amplification using the miniPCR, followed by a DLFD assay.

### 2.7. Comparison of a miniPCR-DLFD and Thick Blood Smear Staining for Microfilariae Detetion

The study population consisted of 10 subjects positive for *B. malayi* mf, 14 subjects positive for *W. bancrofti* mf, and 50 healthy subjects who resided outside endemic areas of LF. Thick blood smear staining for mf detection and miniPCR-DLFD assays were performed using EDTA blood from the study subjects.

## 3. Results

### 3.1. Optimized Condition of Standard PCR Assay for Detection of B. malayi and W. bancrofti

**Figure 3:**
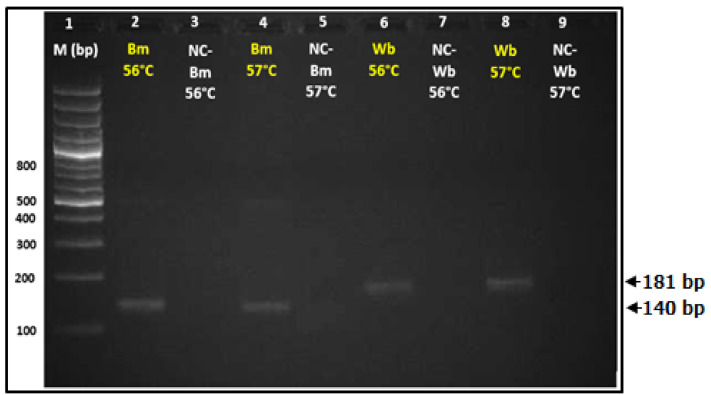
Optimized condition of standard PCR assay for detection of *B. malayi* and *W. bancrofti* DNA. 2% agarose gel electrophoresis showed the amplified *Hha1* gene of *B. malayi* and *Ssp*I gene of *W. bancrofti* at difference annealing temperature (56 °C and 57 °C). Lane 1: 1.5 kb DNA ladder; Lane 2, 4: DNA template of *B. malayi*; Lane 3, 5: negative control of *B. malayi*; Lane 6, 8: DNA template of *W. bancrofti*; Lane 7, 9: negative control of *W. bancrofti*.

The optimal annealing temperature of *Hha*I primer for *B. malayi* and *Ssp*I primer for *W. bancrofti* range between 56–58 °C and 56–57 °C, respectively. The PCR amplification was performed with the annealing temperature of 56 °C and 57 °C. Figure 3 revealed the optimized condition of the standard PCR assay for detection of *B. malayi* and *W. bancrofti* DNA. Two percent agarose gel electrophoresis showed the amplified product of *B. malayi* DNA and amplified product of *W. bancrofti* DNA at different annealing temperatures (56 °C and 57 °C). At both 56 and 57 °C of annealing temperature, the *Hha*I primer amplified 140 bp of the region of the *Hha*I repetitive noncoding DNA sequences of *B. malayi* which showed the product band at 140 bp, whereas the *Ssp*I primer amplified the 181 bp of the region of *W. bancrofti Ssp*I repetitive noncoding DNA sequences showing the product band at 181 bp.

### 3.2. Optimization of Anti-DIG and Streptavidin Concentration

The optimal concentrations of anti-DIG and streptavidin, which showed clearly visible results for test line 1 (*B. malayi*) and test line 2 (*W. bancrofti*) of the DLFD, were the 0.75 µg anti-DIG/strip and 1 µg streptavidin/strip, respectively. The optimal amount of PCR products from the miniPCR that yielded a clear band on the DLFD strip was 1 µL of amplification product from each set of primers (*Hha*I and *Ssp*I) in 100 µL of sample buffer. Clearly detectable, positive pink-colored lines were observed within 10 min.

### 3.3. The miniPCR-DLFD Specificity

**Figure 4:**
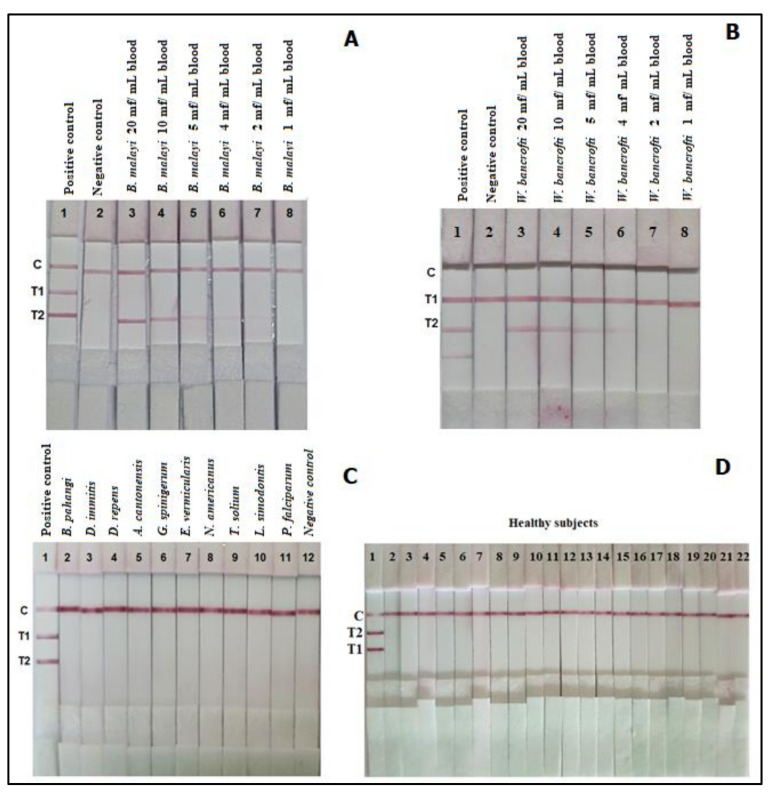
The detection limit of the miniPCR-DLFD using blood samples spiked with various numbers of *B. malayi* mf per mL blood (**A**); *W. bancrofti* mf per mL blood (**B**); and the specificity of the miniPCR-DLFD verified with DNA templates of other parasites (**C**) and DNA templates of healthy subjects (**D**). T1 = detection zone for *Brugia malayi* DNA; T2 = detection zone for *Wuchereria bancrofti* DNA; C = control line.

As illustrated in Figure 4C,D, the DNA from 11 other parasites as well as from blood samples of 50 healthy subjects were all negative in the miniPCR-DLFD assay (Figure 4C). For the sequence similarity of *B. malayi* and *W. bancrofti* primers and other closely related filaria species, there was no significant similarity with any closely related nematode species except that *B. malayi Hha*I repetitive noncoding DNA sequences showed 96.77% identity with *B. timori Hha*I repetitive noncoding DNA sequence (Accession Number AF499118.1).

### 3.4. The Detection Limit of the miniPCR-DLFD

For *B. malayi*, the miniPCR-DLFD still detected a positive band in the samples numbered 17–20, each of which contained 2 mf per milliliter of blood (Figure 4A, Table 1). For *W. bancrofti*, miniPCR-DLFD still detected a positive band in the samples numbered 37–40, each of which contained 4 mf per milliliter of blood (Figure 4B and Table 1).

### 3.5. Comparison of the Giemsa-Stained Thick Blood Smears and the miniPCR-DLFD

**Figure 5:**
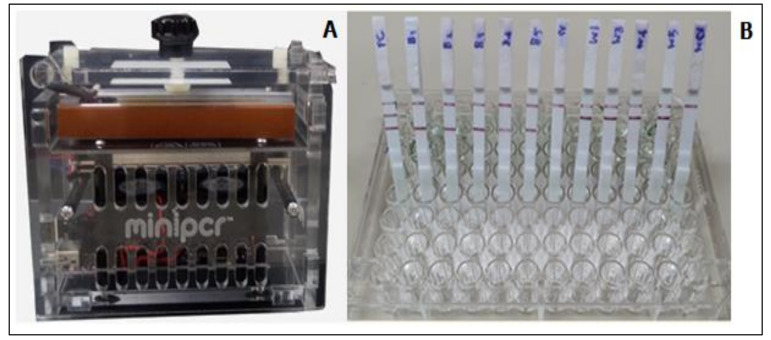
The miniPCR-DLFD for rapid detection and identification of microfilariae of lymphatic filariae in human blood samples. The miniPCR (**A**), and representative DLFD shows reaction of amplicon; strip 1: positive control containing *W. bancrofti* and *B. malayi* DNA, strips 2–11: representative of test samples, and strip 12: negative control (**B**).

The miniPCR showed positive results for all 24 mf positive blood samples and it showed negative results for all 50 mf negative samples from healthy subjects (Figure 5B, Table 2).

## 4. Discussion

In the present study, we developed a miniPCR-DLFD for rapid and visual diagnosis of lymphatic filariasis. The duplex LF detection is another format of lateral flow dipstick that can be used for the detection of more than one target filarial species, and the assay is performed on a strip containing test lines equal to the number of target species to be analyzed [25,26,27].

In laboratory evaluation, both the miniPCR-DLFD and the gold standard thick blood smear staining for mf detection showed concordance in results; thus, it confirms the effectiveness of the miniPCR-DLFD assay for diagnosis of LF infection. 

The detection limit of the miniPCR-DLFD was 2 mf of *B. malayi* per ml of blood sample and 4 mf of *W. bancrofti* per ml of blood. Furthermore, our proposed assay showed no cross-reactivity when testing with the other 11 parasites. Moreover, *Hha*I and *Ssp*I repetitive noncoding DNA sequences showed no significant similarity with other filariae including *B. timori*, *Mansonella* spp., *Loa*, and *Onchocerca volvulus*, except *B. timori* which its *Hha*I amplicons showed 96.77% identity with *B.* *malayi*. This was a limitation of our proposed assay. However, the *B. timori* endemic area is restricted to Timor–Leste and several islands in eastern Indonesia [28]. The developed miniPCR-DLFD may be applied to be used in the other LF endemic areas. 

Zaky et al. used the miniPCR (Amplyus LLC., Cambridge, MA, USA) combined with a lateral flow strip for the rapid detection of amplification products of *Brugia* larvae in mosquitoes, and they highlighted its utility as a backpack-portable point-of-collection diagnostic platform [21]. By comparison, our developed duplex lateral flow dipstick (DLFD) can detect amplification products of both *B. malayi* and *W. bancrofti* microfilariae from human blood samples in a single strip.

These data encourage the usage of the miniPCR-DLFD as an alternative tool for LF detection and it can be used in endemic areas with mixed infections of *B. malayi* and *W. bancrofti*. In addition, in places outside the endemic areas, several cases of lymphatic filariasis have been reported in travelers, military personnel, and expatriates spending time in and returning from the disease-endemic areas, as well as immigrants coming from LF endemic regions. PCR assays can be performed by laboratory personnel who lack the skill of parasite morphology identification [6,29]. 

We have previously developed a semiautomated microfluidic device for rapid higher-throughput detection of microfilariae in animal blood. The microfluidic device trapped the mf from the blood samples within the detection zone of the microfluidic chip. Then, a realtime PCR with high resolution melting analysis (HRM-realtime PCR) was further performed for species identification of the trapped mf [30]. Using the developed platform will eliminate the need for sophisticated thermal cyclers required to perform realtime PCR.

A small portable miniPCR instrument was used in this study to replace the need for a bulkier standard-sized thermocycler. The portable miniPCR from Amplyus used by the current investigation is diminutive in size (5.1 × 12.7 × 10.2 cm) and not heavy (450 g), and it only requires 100–240 V (AC) and 50–60 Hz, 90 W to perform a PCR run of 16 amplifications/round. Furthermore, its cost (~USD 800) is not prohibitive. A WiseSpin microcentrifuge and heat box for DNA extraction are also portable. Regarding processing time, the DNA extraction time for eight samples was 90 min. One run of the miniPCR for eight samples lasts 90 min followed by 15 min of the detection step. Concerning the cost, the reagents for DNA extraction and miniPCR steps cost about USD 3/sample, and one DLFD strip costs USD 2. Thus, the total cost of the miniPCR-DLFD assay is USD 5 per sample. In addition, the storage temperature of the lateral flow dipstick is 4–40 °C, which made it convenient for transportation at ambient temperature. Taken together, a mini PCR amplification platform coupled with a test strip-based detection assay represents a promising diagnostic platform for the diagnosis of LF in a point of collection setting.

## 5. Conclusions

To overcome the impediments to facilitate rapid diagnosis for LF at the collection site associated with infrastructure and expensive equipment, we now propose an alternative method: a miniPCR-DLFD, a reliable user-friendly less labor-intensive detection method of LF diagnosis for point-of-collection.

## Figures and Tables

**Table 1:** The sample ID, number of spiked mf of *B. malayi* and *W. bancrofti* in 1 milliliter (mL) blood sample, concentration of extracted DNA (ng/µL), and results of the miniPCR-DLFD assay.

Sample ID	*B. malayi* mf/mL Blood	Extracted DNA (ng/µL)	miniPCR-DLFD	Sample ID	*W. bancrofti* mf/mL Blood	Extracted DNA (ng/µL)	miniPCR-DLFD
1	20	23	+	25	20	19	+
2	20	22.5	+	26	20	17.5	+
3	20	17.3	+	27	20	17	+
4	20	21	+	28	20	20	+
5	10	22.5	+	29	10	18	+
6	10	22.1	+	30	10	17.5	+
7	10	23	+	31	10	19.5	+
8	10	21.5	+	32	10	17	+
9	5	22.6	+	33	5	19.2	+
10	5	18	+	34	5	21.6	+
11	5	17.7	+	35	5	18.1	+
12	5	23.3	+	36	5	17.9	+
13	4	21.4	+	37	4	16.8	+
14	4	25.7	+	38	4	18.9	+
15	4	21.9	+	39	4	17.5	+
16	4	22.3	+	40	4	17.9	+
17	2	19.2	+	41	2	19.1	−
18	2	21.4	+	42	2	20.6	−
19	2	18.5	+	43	2	18.1	−
20	2	20.4	+	44	2	18.3	−
21	1	27.2	−	45	1	20.2	−
22	1	20.3	−	46	1	16.9	−
23	1	20.8	−	47	1	19.7	−
24	1	21.0	−	48	1	19.3	−

**Table 2:** Comparison of the miniPCR-DLFD based amplification and microfilariae detection by stained thick blood smear for diagnosis of infection with *W. bancrofti* and *B. malayi* in the study blood samples.

Study Sample Group	Number of Study Samples	Thick Blood Smear Staining for Mf Detection		miniPCR-DLFD	
		+	−	+	−
Sample from subject with *B. malayi* mf positive	10	10	0	10	0
Sample from subject with *W. bancrofti* mf positive	14	14	0	14	0
Healthy blood samples	50	0	50	0	50

## Data Availability

Data sharing not applicable.
